# Role of the *IL33 and IL1RL1* pathway in the pathogenesis of Immunoglobulin A vasculitis

**DOI:** 10.1038/s41598-021-95762-5

**Published:** 2021-08-09

**Authors:** Diana Prieto-Peña, Sara Remuzgo-Martínez, Fernanda Genre, Verónica Pulito-Cueto, Belén Atienza-Mateo, Javier Llorca, Belén Sevilla-Pérez, Norberto Ortego-Centeno, Ana Marquez, Leticia Lera-Gómez, María Teresa Leonardo, Ana Peñalba, Javier Narváez, Luis Martín-Penagos, Emilio Rodrigo, José A. Miranda-Filloy, Luis Caminal-Montero, Paz Collado, Javier Sánchez Pérez, Diego de Argila, Esteban Rubio, Manuel León Luque, Juan María Blanco-Madrigal, Eva Galíndez-Agirregoikoa, Oreste Gualillo, Javier Martín, Santos Castañeda, Ricardo Blanco, Miguel A. González-Gay, Raquel López-Mejías

**Affiliations:** 1 Research Group On Genetic Epidemiology and Atherosclerosis in Systemic Diseases and in Metabolic Bone Diseases of the Musculoskeletal System, IDIVAL, Division of Rheumatology, Hospital Universitario Marqués de Valdecilla, Avenida Cardenal Herrera Oria s/n, 39011 Santander, Spain; 2 López Albo´ Post-Residency Programme, Hospital Universitario Marqués de Valdecilla, Santander, Spain; 3 Epidemiology and Computational Biology Department, School of Medicine, Universidad de Cantabria, and CIBER Epidemiología y Salud Pública (CIBERESP), Santander, Spain; 4 Division of Paediatrics, Hospital Universitario San Cecilio, Granada, Spain; 5 Department of Medicine, Universidad de Granada, Granada, Spain; 6 Instituto de Parasitología y Biomedicina ‘López-Neyra’, CSIC, PTS Granada, Granada, Spain; 7 Systemic Autoimmune Disease Unit, Hospital Universitario Clínico San Cecilio, Instituto de Investigación Biosanitaria ibs.GRANADA, Granada, Spain; 8 Division of Paediatrics, Hospital Universitario Marqués de Valdecilla, Santander, Spain; 9 Division of Rheumatology, Hospital Universitario de Bellvitge, Barcelona, Spain; 10 Division of Nephrology, Hospital Universitario Marqués de Valdecilla, IDIVAL-REDINREN, Santander, Spain; 11 Division of Rheumatology, Hospital Universitario Lucus Augusti, Lugo, Spain; 12 Division of Rheumatology, Hospital Universitario Central de Asturias, Instituto de Investigación Sanitaria del Principado de Asturias (ISPA), Oviedo, Spain; 13 Division of Rheumatology, Hospital Universitario Severo Ochoa, Madrid, Spain; 14 Department of Dermatology, Hospital Universitario de La Princesa, Madrid, Spain; 15 Department of Rheumatology, Hospital Universitario Virgen del Rocío, Sevilla, Spain; 16 Department of Rheumatology, Hospital Universitario de Basurto, Bilbao, Spain; 17 SERGAS (Servizo Galego de Saude) and IDIS (Instituto de Investigación Sanitaria de Santiago), NEIRID Lab (Neuroendocrine Interactions in Rheumatology and Inflammatory Diseases), Research Laboratory 9, Hospital Clínico Universitario de Santiago, Santiago de Compostela, Spain; 18 Department of Rheumatology, Hospital Universitario de La Princesa, IIS-Princesa, Madrid, Spain; 19 School of Medicine, Universidad de Cantabria, Santander, Spain; 20 Cardiovascular Pathophysiology and Genomics Research Unit, School of Physiology, Faculty of Health Sciences, University of the Witwatersrand, Johannesburg, South Africa

**Keywords:** Rheumatology, Rheumatic diseases, Vasculitis syndromes

## Abstract

Cytokines signalling pathway genes are crucial factors of the genetic network underlying the pathogenesis of Immunoglobulin-A vasculitis (IgAV), an inflammatory vascular condition. An influence of the *interleukin (IL)33- IL1 receptor like (IL1RL)1* signalling pathway on the increased risk of several immune-mediated diseases has been described. Accordingly, we assessed whether the *IL33-IL1RL1* pathway represents a novel genetic risk factor for IgAV. Three tag polymorphisms within *IL33* (rs3939286, rs7025417 and rs7044343) and three within *IL1RL1* (rs2310173, rs13015714 and rs2058660), that also were previously associated with several inflammatory diseases, were genotyped in 380 Caucasian IgAV patients and 845 matched healthy controls. No genotypes or alleles differences were observed between IgAV patients and controls when *IL33* and *IL1RL1* variants were analysed independently. Likewise, no statistically significant differences were found in *IL33* or *IL1RL1* genotype and allele frequencies when IgAV patients were stratified according to the age at disease onset or to the presence/absence of gastrointestinal (GI) or renal manifestations. Similar results were disclosed when *IL33* and *IL1RL1* haplotypes were compared between IgAV patients and controls and between IgAV patients stratified according to the clinical characteristics mentioned above. Our results suggest that the *IL33-IL1RL1* signalling pathway does not contribute to the genetic network underlying IgAV.

## Introduction

Immunoglobulin-A vasculitis (IgAV), formerly called Henoch-Schönlein purpura (HSP), is an inflammatory small-sized blood vessel disease, more common in children and rarer but more serious in adults^1–3^. The defining pathophysiologic feature of this vasculitis is the IgA1-predominant immune deposits in the vessel walls^1^. Although IgAV usually involves the skin, joints and the gastrointestinal (GI) tract^4–8^, nephritis is also common in affected patients and constitutes the most feared complication of this vasculitis^1–5^. IgAV has a multifactorial aetiology in which genetics plays a relevant role in both the predisposition and clinical course of the disease^6,9,10^. In this regard, outside the human leukocyte antigen region, cytokines signalling pathway genes constitute a key component of the genetic network underlying IgAV^6,11–14^.

Interleukin (IL)-33 is a cytokine that belongs to the IL-1 family^15^. This molecule exerts its biological functions by binding to its receptor, IL-1 receptor like 1 (IL-1RL1) (also known as suppression of tumorigenicity 2 or ST2), a member of the Toll/IL-1 receptor superfamily^16^. This binding leads to the activation of mast cells and Th2 lymphocytes and, consequently, to the production of chemokines, pro-inflammatory and Th2-associated cytokines, as well as increased serum immunoglobulin levels^15^. In accordance with that, a pathogenic role of the IL-33 axis in autoimmunity has been described^17–19^. In addition, several lines of evidence demonstrate that genetic variants located both in the *IL33* and *IL1RL1* genes are implicated in the increased risk of numerous immune-mediated diseases^20–28^.

Taking into account all these considerations, this study aimed to determine, for the first time, the potential implication of the *IL33-IL1RL1* signalling pathway in the pathogenesis of IgAV. For this purpose, we genotyped three tag genetic variants within *IL33* and three tag polymorphisms within *IL1RL1*, which cover the major variability of these *loci* and that were previously associated with several inflammatory diseases, in the largest series of Caucasian IgAV patients ever assessed for genetic studies.

## Patients and methods

### Study population

A series of 380 unrelated Spanish patients of European ancestry who fulfilled Michel et al*.* criteria^29^ and/or the American College of Rheumatology classification criteria^30^ for IgAV-HSP and/or the 2012 revised International Chapel Hill Consensus Conference Nomenclature^31^ definition for IgAV were included in the present study. Centres involved in the recruitment of these patients included Hospital Universitario Marqués de Valdecilla (Santander), Hospital Universitario San Cecilio (Granada), Hospital Universitario de Bellvitge (Barcelona), Hospital Universitario Lucus Augusti (Lugo), Hospital Universitario Central de Asturias (Oviedo), Hospital Universitario Severo Ochoa and Hospital Universitario de La Princesa (Madrid), Hospital Universitario Virgen del Rocío (Sevilla) and Hospital Universitario de Basurto (Bilbao). Information on the main clinical features of these patients is displayed in Table 1. For GI manifestations, bowel angina was considered present if there was diffuse abdominal pain that worsened after meals or bowel ischemia usually with bloody diarrhoea. GI bleeding was defined as the presence of melena, haematochezia, or a positive test for occult blood in the stool. Renal manifestations were defined to be present if at least one of the following findings was observed: haematuria, proteinuria, or nephrotic syndrome at any time over the clinical course of the disease and/or renal sequelae (persistent renal involvement) at last follow-up.

**Table 1:** Main clinical features of the 380 patients with IgAV included in the study.

	% (n)
Children (age ≤ 20 years)/ adults (age > 20 years) (n)	294/86
Percentage of females	47.9
Age at disease onset (years, median [IQR])	7(5–19)
Duration of follow-up (years, median [IQR])	1 (1–3)
Palpable purpura and/or maculopapular rash	100 (380)
Arthralgia and/or arthritis	52.9 (201)
GI manifestations (if “a” and/or “b”)	53.2 (202)
(a) Bowel angina	50.5 (192)
(b) GI bleeding	16.3 (62)
Renal manifestations (if any of the following characteristics)	36.1 (137)
(a) Haematuria*	34.5 (131)
(b) Proteinuria*	32.9 (125)
(c) Nephrotic syndrome*	5.5 (21)
(d) Renal sequelae (persistent renal involvement) **	6.6 (25)

IgAV: IgA vasculitis; IQR: interquartile range; GI: gastrointestinal.

*At any time over the clinical course of the disease.

**At last follow-up.

In addition, a set of 845 sex and ethnically matched healthy controls without history of cutaneous vasculitis or any other autoimmune disease, constituted by blood donors from Hospital Universitario Marqués de Valdecilla (Santander) and National DNA Bank Repository (Salamanca), was also included in this study.

For experiments involving humans and the use of human blood samples, all the methods were carried out in accordance with the approved guidelines and regulations, according to the Declaration of Helsinki. All experimental protocols were approved by the Ethics Committees of clinical research of Cantabria (for Hospital Universitario Marqués de Valdecilla, Santander), Ethics Committee of clinical research of Granada (for Hospital Universitario San Cecilio, Granada), Ethics Committee of clinical research of Barcelona (for Hospital Universitario de Bellvitge, Barcelona), Ethics Committee of clinical research of Galicia (for Hospital Universitario Lucus Augusti, Lugo), Ethics Committee of clinical research of Asturias (for Hospital Universitario Central de Asturias, Oviedo), Ethics Committee of clinical research of Madrid (for Hospital Universitario Severo Ochoa and Hospital Universitario de La Princesa, Madrid), Ethics Committee of clinical research of Sevilla (for Hospital Universitario Virgen del Rocío, Sevilla) and Ethics Committee of clinical research of Euskadi (for Hospital Universitario de Basurto, Bilbao). Patients with IgAV and healthy controls signed an informed written consent before being included in the study.

### Single nucleotide polymorphisms selection and genotyping methods

Three tag genetic variants within *IL33* (rs3939286, rs7025417 and rs7044343) and three tag polymorphisms within *IL1RL1* (rs2310173, rs13015714 and rs2058660) were selected in this study. Regarding *IL33*, rs3939286 is situated in an intergenic region close to *IL33,* whereas rs7025417 and rs7044343 are located in the promoter and in intron 5 of the gene, respectively. With respect to *IL1RL1*, both rs2310173 and r2058660 are placed close to *IL1RL1,* while rs13015714 is downstream of the gene. Interestingly, these six polymorphisms map and tag both *IL33* and *IL1RL1* regions as previously described^20^. In addition, the *IL33* and *IL1RL1* genetic variants selected in our study were previously associated with several immune-mediated diseases^20–28^. In particular, *IL33* rs3939286 was previously implicated in the development of subclinical atherosclerosis in patients with rheumatoid arthritis (RA)^20^ as well as in the pathogenesis of inflammatory bowel (IBD) disease^21^ and asthma^22^, whereas *IL33* rs7025417 was previously associated with coronary artery disease^23^, and rs7044343 with RA^24^. Regarding *IL1RL1*, a relevant role of rs2310173 in ankylosing spondylitis^25^ and ulcerative colitis^26^, as well as an association of rs13015714 with celiac disease ^27^ and IBD^21^, and an influence of rs2058660 on the development of Crohn’s disease^28^ were previously reported.

Genomic deoxyribonucleic acid (DNA) from all the individuals included in the study was extracted from peripheral blood using standard procedures.

Patients with IgAV and healthy controls were genotyped for the six polymorphisms mentioned above using predesigned TaqMan 5′ single-nucleotide polymorphism genotyping assays (C___2762168_10 for rs3939286, C__31940410_20 for rs7025417, C__29340326_10 for rs7044343, C___2676437_10 for rs2310173, C__31439507_10 for rs13015714 and C__11487892_10 for rs2058660).

Genotyping was performed in a QuantStudioTM 7 Flex Real-Time polymerase chain reaction system, according to the conditions recommended by the manufacturer (Applied Biosystems, Foster City, CA, USA).

Negative controls and duplicate samples were included to check the accuracy of the genotyping.

### Statistical analyses

Genotype data were checked for deviation from Hardy–Weinberg equilibrium (HWE).

Differences in *IL33* and *IL1RL1* frequencies were evaluated between patients with IgAV and healthy controls and between patients with IgAV stratified according to specific clinical characteristics of the disease (age at disease onset or presence/absence of GI or renal manifestations).

First, each *IL33* and *IL1RL1* polymorphism was analysed independently. Both genotype and allele frequencies were calculated and compared between the groups mentioned above by chi-square test. Strength of association was estimated using odds ratios (OR) and 95% confidence intervals (CI).

Then, allelic combinations (haplotypes) of both *IL33* and *IL1RL1* polymorphisms were carried out. Haplotype frequencies were calculated by the Haploview v4.2 software (http://broad.mit.edu/mpg/haploview) and then compared between the groups mentioned above by chi-square test. Strength of association was estimated by OR and 95% CI.

*P*-values were two-tailed and those lower than 0.05 were considered as statistically significant. All analyses were performed with the STATA statistical software 12/SE (Stata Corp., College Station, TX, USA).

## Results

### Genotyping quality control

*IL33* rs3939286, *IL33* rs7025417, *IL33* rs7044343, *IL1RL1* rs2310173, *IL1RL1* rs13015714 and *IL1RL1* rs2058660 were in HWE.

The genotyping success rate for each polymorphism evaluated in this study was 99.9% for *IL33* rs3939286, 99.4% for *IL33* rs7025417, 99.8% for *IL33* rs7044343, 100% for *IL1RL1* rs2310173, 99.6% for *IL1RL1* rs13015714 and 99.8% for *IL1RL1* rs2058660.

Genotype and allele frequencies of each *IL33* and *IL1RL1* polymorphism were similar to that reported for populations of European origin in the 1000 Genomes Project (http://www.internationalgenome.org/).

### Differences in *IL33* and *IL1RL1* genotype and allele frequencies between patients with IgAV and healthy controls

In a first step, we compared genotype and allele frequencies of each *IL33* and *IL1RL1* variant assessed independently between patients with IgAV and healthy controls.

As shown in Table 2, similar genotype and allele *IL33* and *IL1RL1* frequencies were observed in patients with IgAV when compared to healthy controls (Table 2).

**Table 2:** Genotype and allele frequencies of *IL33* and *IL1RL1* genes in patients with IgAV and healthy controls.

Change				Genotypes, % (n)			Alleles, % (n)	
Locus	SNP	1/2	Samples set	1/1	1/2	2/2	1	2
*IL33*	rs3939286	C/T	IgAV patients	49.1 (186)	40.9 (155)	10.0 (38)	69.5 (527)	30.5 (231)
			Healthy controls	49.0 (414)	41.4 (350)	9.6 (81)	69.7 (1178)	30.3 (512)
*IL33*	rs7025417	T/C	IgAV patients	68.1 (254)	29.5 (110)	2.4 (9)	82.8 (618)	17.2 (128)
			Healthy controls	70.8 (598)	25.9 (219)	3.3 (28)	83.7 (1415)	16.3 (275)
*IL33*	rs7044343	T/C	IgAV patients	42.3 (160)	42.1 (159)	15.6 (59)	63.4 (479)	36.6 (277)
			Healthy controls	44.5 (376)	43.9 (371)	11.6 (98)	66.4 (1123)	33.6 (567)
*IL1RL1*	rs2310173	G/T	IgAV patients	29.2 (111)	46.1 (175)	24.7 (94)	52.2 (397)	47.8 (363)
			Healthy controls	30.2 (255)	46.7 (395)	23.1 (195)	53.6 (905)	46.4 (785)
*IL1RL1*	rs13015714	T/G	IgAV patients	56.3 (211)	39.5 (148)	4.3 (16)	76.0 (570)	24.0 (180)
			Healthy controls	57.2 (483)	37.2 (314)	5.7 (48)	75.7 (1280)	24.3 (410)
*IL1RL1*	rs2058660	A/G	IgAV patients	56.9 (215)	38.6 (146)	4.5 (17)	76.2 (576)	23.8 (180)
			Healthy controls	56.7 (479)	37.5 (317)	5.8 (49)	75.4 (1275)	24.6 (415)

IgAV: IgA vasculitis; SNP: single nucleotide polymorphism.

No statistically significant differences in the *IL33* and *IL1RL1* genotype and allele frequencies were disclosed when patients with IgAV were compared to healthy controls (*p* ≥ 0.05 in all the cases).

### Differences in *IL33* and *IL1RL1* genotype and allele frequencies between patients with IgAV stratified according to specific clinical characteristics of the disease

Then, potential differences in the genotype and allele frequencies of each *IL33* and *IL1RL1* genetic variant were evaluated between patients with IgAV stratified according to specific clinical characteristics of the disease.

Given that IgAV is often a benign and self-limited pathology in children and a more severe condition in adults, we assessed if potential differences in *IL33* and *IL1RL1* could exist in IgAV patients stratified according to the age at disease onset. With this respect, and as shown in Table 3, similar genotype and allele *IL33* and *IL1RL1* frequencies were disclosed when children (age ≤ 20 years) were compared to adults (age > 20 years) (Table 3).

**Table 3:** Genotype and allele frequencies of *IL33* and *IL1RL1* genes in patients with IgAV stratified according to clinical characteristics of the disease.

Polymorphism	Children (Age ≤ 20 years)		GI manifestations		Renal manifestations	
	Yes (n = 294)	No (n = 86)	Yes (n = 202)	No (n = 178)	Yes (n = 137)	No (n = 243)
***IL33*rs3939286**						
CC	47.8 (140)	53.5 (46)	47.3 (95)	51.1 (91)	48.5 (66)	49.4 (120)
CT	42.0 (123)	37.2 (32)	42.3 (85)	39.3 (70)	39.7 (54)	41.6 (101)
TT	10.2 (30)	9.3 (8)	10.4 (21)	9.6 (17)	11.8 (16)	9.1 (22)
C	68.8 (403)	72.1 (124)	68.4 (275)	70.8 (252)	68.4 (186)	70.2 (341)
T	31.2 (183)	27.9 (48)	31.6 (127)	29.2 (104)	31.6 (86)	29.8 (145)
***IL33*rs7025417**						
TT	66.6 (191)	73.3 (63)	68.2 (135)	68.0 (119)	69.9 (93)	67.1 (161)
TC	31.0 (89)	24.4 (21)	28.8 (57)	30.3 (53)	27.1 (36)	30.8 (74)
CC	2.4 (7)	2.3 (2)	3.0 (6)	1.7 (3)	3.0 (4)	2.1 (5)
T	82.1 (471)	85.5 (147)	82.6 (327)	83.1 (291)	83.5 (222)	82.5 (396)
C	17.9 (103)	14.5 (25)	17.4 (69)	16.9 (59)	16.5 (44)	17.5 (84)
***IL33*rs7044343**						
TT	44.2 (129)	36.0 (31)	43.9 (88)	40.7 (72)	44.5 (61)	41.1 (99)
TC	39.4 (115)	51.2 (44)	39.8 (80)	44.6 (79)	39.4 (54)	43.6 (105)
CC	16.4 (48)	12.8 (11)	16.4 (33)	14.7 (26)	16.1 (22)	15.4 (37)
T	63.9 (373)	61.6 (106)	63.7 (256)	63.0 (223)	64.2 (176)	62.9 (303)
C	36.1 (211)	38.4 (66)	36.3 (146)	37.0 (131)	35.8 (98)	37.1 (179)
***IL1RL1*rs2310173**						
GG	29.6 (87)	27.9 (24)	30.2 (61)	28.1 (50)	32.1 (44)	27.6 (67)
GT	45.2 (133)	48.8 (42)	45.0 (91)	47.2 (84)	43.1 (59)	47.7 (116)
TT	25.2 (74)	23.3 (20)	24.8 (50)	24.7 (44)	24.8 (34)	24.7 (60)
G	52.2 (307)	52.3 (90)	52.7 (213)	51.7 (184)	53.6 (147)	51.4 (250)
T	47.8 (281)	47.7 (82)	47.3 (191)	48.3 (172)	46.4 (127)	48.6 (236)
***IL1RL1*rs13015714**						
TT	55.5 (161)	58.8 (50)	57.3 (114)	55.1 (97)	61.8 (84)	53.1 (127)
TG	40.0 (116)	37.6 (32)	40.2 (80)	38.6 (68)	33.8 (46)	42.7 (102)
GG	4.5 (13)	3.5 (3)	2.5 (5)	6.3 (11)	4.4 (6)	4.2 (10)
T	75.5 (438)	77.6 (132)	77.4 (308)	74.4 (262)	78.7 (214)	74.5 (356)
G	24.5 (142)	22.4 (38)	22.6 (90)	25.6 (90)	21.3 (58)	25.5 (122)
***IL1RL1*rs2058660**						
AA	56.3 (165)	58.8 (50)	57.9 (117)	55.7 (98)	62.2 (84)	53.9 (131)
AG	39.2 (115)	36.5 (31)	39.1 (79)	38.1 (67)	31.9 (43)	42.4 (103)
GG	4.4 (13)	4.7 (4)	3.0 (6)	6.3 (11)	5.9 (8)	3.7 (9)
A	75.9 (445)	77.1 (131)	77.5 (313)	74.7 (263)	78.1 (211)	75.1 (365)
G	24.1 (141)	22.9 (39)	22.5 (91)	25.3 (89)	21.9 (59)	24.9 (121)

IgAV: IgA vasculitis; GI: gastrointestinal.

No statistically significant differences in the *IL33* and *IL1RL1* genotype and allele frequencies were disclosed between patients with IgAV stratified according to the age at disease onset, the presence/absence of GI or renal manifestations (*p* ≥ 0.05 in all the cases).

In addition, we compared genotype and allele frequencies of *IL33* and *IL1RL1* in patients with IgAV stratified according to the presence/absence of GI or renal manifestations. Accordingly, similar genotype and allele *IL33* and *IL1RL1* frequencies were disclosed when patients with IgAV who developed GI manifestations were compared to those who did not exhibit these complications (Table 3). This was also the case when patients with IgAV were stratified according to the presence/absence of renal manifestations (Table 3).

### Haplotype analyses of *IL33* and *IL1RL1*

In a further step, we also examined whether haplotype frequencies of both *IL33* and *IL1RL1* differed between patients with IgAV and healthy controls as well as between patients with IgAV stratified according to the specific clinical characteristics of the disease mentioned above.

The haplotype analysis did not yield additional information since *IL33* and *IL1RL1* haplotypes frequencies were similar in patients with IgAV when compared to healthy controls (Table 4). This was also the case when patients with IgAV were stratified according to the age at disease onset and the presence/absence of GI or renal manifestations (Supplementary Tables S1–S3 online).

**Table 4:** Haplotype analysis of *IL33* and *IL1RL1* genes between patients with IgAV and healthy controls.

***IL33*haplotypes**			*p*	OR (95% CI)
rs3939286	rs7025417	rs7044343		
C	T	T	–	Ref
C	T	C	0.26	1.15 (0.89–1.47)
T	T	T	0.77	1.04 (0.78–1.40)
T	T	C	0.35	1.15 (0.85–1.58)
T	C	T	0.84	1.04 (0.68–1.58)
C	C	T	0.53	1.14 (0.74–1.71)

***IL1RL1*haplotypes**			*p*	OR (95% CI)
rs2310173	rs13015714	rs2058660		
G	T	A	–	Ref
T	T	A	0.10	1.19 (0.96–1.46)
T	G	G	0.50	0.91 (0.70–1.19)
G	G	G	0.16	1.26 (0.90–1.77)

IgAV: IgA vasculitis; OR: Odds Ratio; CI: confidence interval.

Haplotypes of *IL33* and *IL1RL1* with a frequency higher than 5% are shown.

## Discussion

Several pieces of evidence clearly indicate that a common genetic background may underlie different inflammatory diseases^32,33^. In this regard, the *IL33-IL1RL1* signalling pathway is described as a shared genetic risk factor for numerous immune-related diseases^20–28^.

Based on these considerations, in this study we aimed to determine whether the *IL33-IL1RL1* signalling pathway is also implicated in the pathogenesis of IgAV, a small-vessel vasculitis in which cytokines signalling pathway genes are considered as an essential component in its etiology^6,11–14^. For that purpose, six polymorphisms within the *IL33-IL1RL1* signalling pathway, which cover most of the variability of both *IL33* and *IL1RL1* regions and that were previously related to numerous immune-mediated diaseases^20–28^, were evaluated in the largest series of Caucasian patients with IgAV ever assessed for genetic studies. Our results showed no influence of the *IL33-IL1RL1* signalling pathway on the susceptibility to IgAV when we studied each of the polymorphisms separately. Moreover, when we analysed *IL33* genetic variants as well as *IL1RL1* polymorphisms together conforming haplotypes, our results revealed a lack of association between the *IL33-IL1RL1* signalling pathway and IgAV susceptibility. Furthermore, no specific association of *IL33* and *IL1RL1* polymorphisms (assessed independently or combined conforming haplotypes) with clinical features of IgAV was observed in our study, indicating that this pathway does not represent a risk factor for the severity of the disease.

Data on the potential effect of the *IL33-IL1RL1* signalling pathway on vasculitis were scarce so far. A study performed in Caucasians evaluated the role of this pathway in the susceptibility to giant cell arteritis (GCA)^34^, another primary systemic vasculitis that involves large and middle-sized blood vessels^31,35^. In keeping with our results, no influence of *IL33* rs3939286, *IL33* rs7044343, *IL1RL1* rs2310173, *IL1RL1* rs13015714 and *IL1RL1* rs2058660 on GCA was detected^34^. By contrast, a consistent association between *IL33* rs7025417 and GCA was found, pointing out this polymorphism as a novel genetic risk factor for GCA susceptibility^34^. In addition, the potential role of an *IL33* polymorphism (rs1342326) in the pathogenesis of Behçet’s disease, a condition affecting blood vessels of all sizes and types^31^, was assessed in Iranians^36^. With this respect, an effect of this genetic variant in the susceptibility to this disease was disclosed^36^.

The results derived from our study are of potential clinical relevance. Vasculitides comprises a heterogeneous group of autoimmune diseases that often have overlapping clinical and pathological manifestations^37^. Although their complex aetiology is far from being completely understood, differences among them in molecular terms have been postulated^38^. Our data support this hypothesis since no implication of the *IL33-IL1RL1* signalling pathway in the pathogenesis of IgAV in Caucasians was disclosed, unlike GCA^34^.

In summary, based on a large series of Caucasian patients, our results suggest that the *IL33-IL1RL1* signalling pathway does not contribute to the genetic network underlying IgAV.

## Supplementary Information

Supplementary Information.
