# Supplementary material for: Role of the IL33 and IL1RL1 pathway in the pathogenesis of Immunoglobulin A vasculitis
Source: Sci Rep. 2021 Aug 9;11:16163. doi: 10.1038/s41598-021-95762-5 (PMC8352942; doi:10.1038/s41598-021-95762-5)
Supplement: Supplementary file 1 — Supplementary Information. [file 41598_2021_95762_MOESM1_ESM.doc]

**Role of the *IL33 and IL1RL1* pathway in the pathogenesis of Immunoglobulin A vasculitis**

Diana Prieto-Peña^1±^, Sara Remuzgo-Martínez^1±^, Fernanda Genre^1±^, Verónica Pulito-Cueto^1^, Belén Atienza-Mateo^1,2^, Javier Llorca^3^, Belén Sevilla-Pérez^4^, Norberto Ortego-Centeno^5^, Ana Marquez^6,7^, Leticia Lera-Gómez^1^, María Teresa Leonardo^8^, Ana Peñalba^8^, Javier Narváez^9^, Luis Martín-Penagos^10^, Emilio Rodrigo^10^, José A. Miranda-Filloy^11^, Luis Caminal-Montero^12^, Paz Collado^13^, Javier Sánchez Pérez^14^, Diego de Argila^14^, Esteban Rubio^15^, Manuel León Luque^15^, Juan María Blanco-Madrigal^16^, Eva Galíndez-Agirregoikoa^16^, Oreste Gualillo^17^, Javier Martín^6^, Santos Castañeda^18^, Ricardo Blanco^1^, Miguel A. González-Gay^1, 19, 20§^, Raquel López-Mejías^1§*^

| **Supplementary Table S1.** Haplotype analysis of *IL33* and *IL1RL1* genes in patients with IgAV stratified according to the age at disease onset. | | | | |
| --- | --- | --- | --- | --- |
| *IL33* haplotypes | | | p | OR [95% CI] |
| rs3939286 | rs7025417 | rs7044343 |  |  |
| C | T | T | - | Ref. |
| C | T | C | 0.64 | 0.90 [0.55-1.47] |
| T | T | T | 0.55 | 1.19 [0.65-2.27] |
| T | T | C | 0.87 | 1.05 [0.57-1.98] |
| T | C | T | 0.68 | 1.19 [0.50-3.13] |
| C | C | T | 0.20 | 1.79 [0.71-5.42] |
|  |  |  |  |  |
| *IL1RL1* haplotypes | | | p | OR [95% CI] |
| rs2310173 | rs13015714 | rs2058660 |  |  |
| G | T | A | - | Ref. |
| T | T | A | 0.90 | 1.03 [0.68-1.56] |
| T | G | G | 0.71 | 1.11 [0.63-1.99] |
| G | G | G | 0.55 | 1.22 [0.62-2.59] |
| IgAV: IgA vasculitis; OR: Odds Ratio; CI: confidence interval.  Haplotypes of *IL33* and *IL1RL1* with a frequency higher than 5% are shown. | | | | |

| **Supplementary Table S2.** Haplotype analysis of *IL33* and *IL1RL1* genes in patients with IgAV stratified according to the presence/absence of GI manifestations. | | | | |
| --- | --- | --- | --- | --- |
| *IL33* haplotypes | | | p | OR [95% CI] |
| rs3939286 | rs7025417 | rs7044343 |  |  |
| C | T | T | - | Ref. |
| C | T | C | 0.87 | 0.97 [0.63-1.48] |
| T | T | T | 0.84 | 1.05 [0.63-1.75] |
| T | T | C | 0.54 | 1.17 [0.69-1.97] |
| T | C | T | 0.51 | 1.25 [0.90-2.66] |
| C | C | T | 0.63 | 1.18 [0.57-2.47] |
|  |  |  |  |  |
| *IL1RL1* haplotypes | | | p | OR [95% CI] |
| rs2310173 | rs13015714 | rs2058660 |  |  |
| G | T | A | - | Ref. |
| T | T | A | 0.56 | 0.90 [0.64-1.28] |
| T | G | G | 0.36 | 0.82 [0.51-1.30] |
| G | G | G | 0.25 | 0.73 [0.41-1.29] |
| IgAV: IgA vasculitis; GI: gastrointestinal; OR: Odds Ratio; CI: confidence interval.  Haplotypes of *IL33* and *IL1RL1* with a frequency higher than 5% are shown. | | | | |

| **Supplementary Table S3.** Haplotype analysis of *IL33* and *IL1RL1* genes in patients with IgAV stratified according to the presence/absence of renal manifestations. | | | | |
| --- | --- | --- | --- | --- |
| *IL33* haplotypes | | | p | OR [95% CI] |
| rs3939286 | rs7025417 | rs7044343 |  |  |
| C | T | T | - | Ref. |
| C | T | C | 0.74 | 0.93 [0.59-1.45] |
| T | T | T | 0.99 | 1.00 [0.59-1.70] |
| T | T | C | 0.76 | 1.08 [0.63-1.84] |
| T | C | T | 0.83 | 0.93 [0.42-1.97] |
| C | C | T | 0.62 | 1.19 [0.56-2.44] |
|  |  |  |  |  |
| *IL1RL1* haplotypes | | | p | OR [95% CI] |
| rs2310173 | rs13015714 | rs2058660 |  |  |
| G | T | A | - | Ref. |
| T | T | A | 0.92 | 0.98 [0.68-1.41] |
| T | G | G | 0.07 | 0.63 [0.37-1.06] |
| G | G | G | 0.92 | 0.97 [0.53-1.74] |
| IgAV: IgA vasculitis; OR: Odds Ratio; CI: confidence interval.  Haplotypes of *IL33* and *IL1RL1* with a frequency higher than 5% are shown. | | | | |
